# Improving the therapeutic efficacy of mesenchymal stromal cells to restore perfusion in critical limb ischemia through pulsed focused ultrasound

**DOI:** 10.1038/srep41550

**Published:** 2017-02-07

**Authors:** Pamela A. Tebebi, Saejeong J. Kim, Rashida A. Williams, Blerta Milo, Victor Frenkel, Scott R. Burks, Joseph A. Frank

**Affiliations:** 1 Frank Lab, Radiology and Imaging Sciences Dept., Clinical Center, National Institutes of Health, Bethesda, Maryland, USA; 2 Department of Biomedical Engineering, Catholic University of America, Washington, DC, USA; 3 Department of Diagnostic Radiology and Nuclear Medicine, University of Maryland School of Medicine, Baltimore, MD, USA; 4 National Institutes of Biomedical Imaging and Bioengineering, National Institutes of Health, Bethesda, Maryland, USA

## Abstract

Mesenchymal stem cells (MSC) are promising therapeutics for critical limb ischemia (CLI). Mechanotransduction from pulsed focused ultrasound (pFUS) upregulates local chemoattractants to enhance homing of intravenously (IV)-infused MSC and improve outcomes. This study investigated whether pFUS exposures to skeletal muscle would improve local homing of iv-infused MSCs and their therapeutic efficacy compared to iv-infused MSCs alone. CLI was induced by external iliac arterial cauterization in 10–12-month-old mice. pFUS/MSC treatments were delayed 14 days, when surgical inflammation subsided. Mice were treated with iv-saline, pFUS alone, IV-MSC, or pFUS and IV-MSC. Proteomic analyses revealed pFUS upregulated local chemoattractants and increased MSC tropism to CLI muscle. By 7 weeks post-treatment, pFUS + MSC significantly increased perfusion and CD31 expression, while reducing fibrosis compared to saline. pFUS or MSC alone reduced fibrosis, but did not increase perfusion or CD31. Furthermore, MSCs homing to pFUS-treated CLI muscle expressed more vascular endothelial growth factor (VEGF) and interleukin-10 (IL-10) than MSCs homing to non-pFUS-treated muscle. pFUS + MSC improved perfusion and vascular density in this clinically-relevant CLI model. The molecular effects of pFUS increased both MSC homing and MSC production of VEGF and IL-10, suggesting microenvironmental changes from pFUS also increased potency of MSCs *in situ* to further enhance their efficacy.

Peripheral artery disease (PAD) arises from limited or restricted blood flow that can lead to limb pain, disability, or loss^1^,^2^,^3^. Critical limb ischemia (CLI) is the most serious form of PAD. It is characterized by severely diminished quality of life and carries greater risks of amputation, nonfatal ischemic events, and death, yet few treatment options exist^4^,^5^,^6^,^7^,^8^. Treatment is often ineffective or unfeasible clinically. Biologics such as gene or cell therapy have potential to improve quality of life and treat the underlying disease^7^,^9^,^10^,^11^,^12^. Many preclinical cell therapy studies demonstrate improved perfusion of ischemic limbs after intravenous (IV), intra-arterial (IA), or intramuscular (IM) injection of various stem cells types^3^,^13^,^14^ in a number of different experimental models^15^,^16^,^17^,^18^,^19^,^20^,^21^,^22^. Clinical cell therapy trials however, have had varied success. While some have shown wound healing, improved perfusion of lower extremities, reduced pain^12^, and less need for amputation^11^,^23^, many trials did not demonstrate any clinical benefit^24^,^25^,^26^,^27^.

Mesenchymal stromal cells (MSC), also known as mesenchymal stem cells, migrate toward and proliferate in response to, chemokine or cytokine gradients at sites of ischemia or inflammation^28^. MSCs promote regeneration of damaged tissue, reduce inflammation, and stimulate angiogenesis^29^,^30^. However, only a small fraction of injected cells (<1–3%) home and can be found in the affected parenchyma^31^. We have shown that image-guided pulsed focused ultrasound (pFUS) increases local expression of cytokines, chemokines, trophic factors (CCTF), and cell adhesion molecules (CAM) in normal and diseased tissues^32^,^33^,^34^,^35^,^36^. The molecular responses to pFUS can be harnessed for enhanced homing permeability and retention (EHPR) of infused MSC to pFUS-targeted sites^32^,^33^,^34^,^35^,^36^. We have demonstrated that pFUS to kidneys prior to IV MSC infusions significantly improved survival and renal function in established acute kidney injury (AKI)^32^. In that study, we also observed that MSC homing to pFUS-treated kidneys expressed more interleukin (IL)-10 than MSC homing to non-pFUS-treated kidneys, suggesting that molecular responses to pFUS altered MSC physiology in addition to increasing tropism to sonicated tissues.

This study investigated whether pFUS sonication to ischemic muscle in conjunction with IV MSC infusions would improve limb perfusion compared to IV MSC injections alone in a CLI model using aged mice. External iliac arteries (EIA) were unilaterally excised in female C3H mice to induce CLI. Mice were aged 10–12 months to reflect the clinical population affected by CLI (50–65+ years old). pFUS and/or MSC treatment was performed 14 days after EIA surgery to allow surgically-induced inflammation to subside. Laser Doppler perfusion imaging (LDPI) was performed over 7 weeks and mice were then euthanized for histological evaluation. We also investigated whether MSC expression of beneficial cytokines or growth factors was altered after pFUS treatment by immunostaining for human IL-10 and vascular endothelial growth factor (VEGF).

## Results

**Figure 1:**
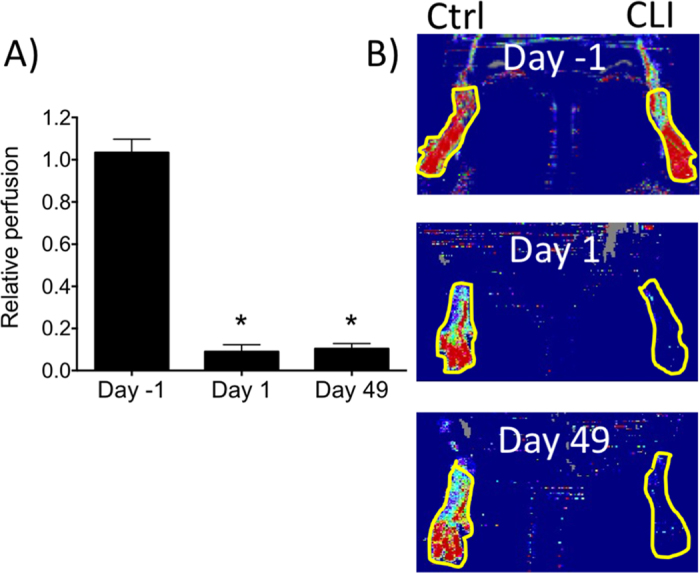
Laser Doppler perfusion imaging (LDPI) of feet in critical limb ischemia (CLI) up to 7 weeks post-surgery. (**A**) Relative limb perfusion in healthy (Day-1) legs and ischemic legs at 1 day after surgery (Day 1) and at 7 weeks post-surgery (Day 49) (n = 8) (perfusion in ischemic legs is normalized to perfusion of the contralateral leg) (**B**) Representative LDPI at days -1, 1, and 49 showing regions-of-interest for quantification.

To induce appropriately severe CLI, the EIAs of 10–12-month-old mice were double ligated and cauterized. Blood flow was significantly (p ≤ 0.001) decreased by approximately 85% compared to normal contralateral limbs and limb remained similarly hypoperfused over 7 weeks (Fig. 1). Based on this model, we investigated the natural proteomic history of surgically-induced CLI and additional proteomic changes after pFUS to determine the effects of sonication on the CLI muscle microenvironment.

### Proteomic response following CLI surgery and pFUS

**Figure 2:**
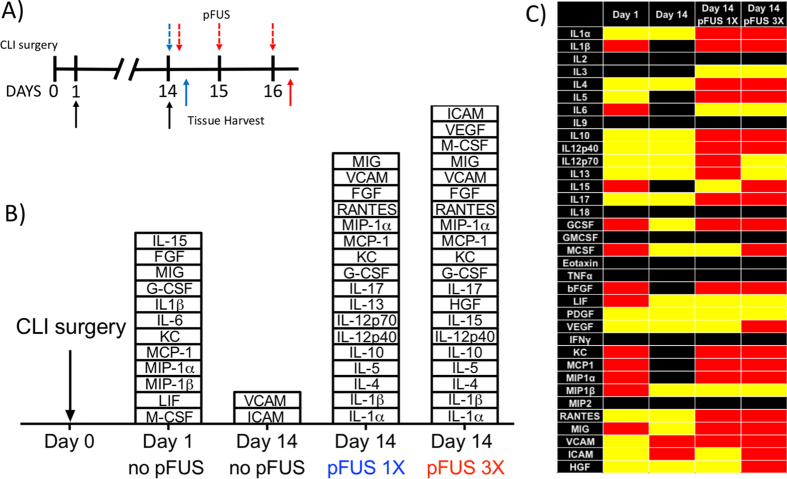
Proteomic responses to critical limb ischemia (CLI) alone and pulsed focused ultrasound (pFUS) in CLI skeletal muscle. (**A**) Experimental timeline showing CLI at Day 0. pFUS treatment time points (Days 14, 15, and 16) are indicated by dashed arrows on top of timeline and tissue harvest time points (Days 1, 14, and 16) are indicated by solid arrows on the bottom of timeline. (**B**) Stack box plot showing significant (p < 0.05; n = 6 mice per group per time point) elevations of cytokines, chemokines, trophic factors, and cell adhesion molecules compared to normal muscle after CLI alone (Days 1 and 14), after 1 course of pFUS (day 14 pFUS 1x), and after 3 daily courses of pFUS (day 14 pFUS 3x). Comparisons were made using one-way ANOVA with Bonferroni corrections; n = 6 mice/time point. (**C**) Heat map depicting significant differences shown in (**B**). Red indicates significant elevations, yellow represents no change, and black indicates undetectable protein levels. (see Supplemental Figure 1 for primary data and Supplemental Table 1 for abbreviations).

We previously showed that pFUS to skeletal muscle increased CCTF and CAM in <12-week-old mice that returned to baseline by ~48 hr post-pFUS^33^,^37^. In the current study, hamstrings were sonicated to obtain sufficient material for proteomic and histological analyses. Proteomic analyses of CLI muscle are summarized in Fig. 2. Analyses were performed before surgery (Day 0 sham control), days 1 and 14 post-surgery to assess the natural history of CLI. Protein levels from all three groups were compared by a one-way ANOVA and significant (p < 0.05) increases of many CCTF were observed at 1 day post-surgery compared to the Day 0 sham. These include: Interleukins (IL)-1β, -6, -15, monokine-induced by gamma interferon (MIG), granulocyte colony stimulating factor (G-CSF), macrophage colony stimulating factor (M-CSF), monocyte chemoattractant protein-1 (MCP-1), macrophage inflammatory protein-1α and -1β (MIP-1α and MIP-1β), leukemic inducible factor (LIF), keratinocyte chemoattractant (KC [CXCL1]), and fibroblast growth factor (FGF) (Fig. 2). Fourteen days post-surgery, only vascular cell adhesion molecule (VCAM) and intercellular adhesion molecule (ICAM) were significantly elevated in muscle compared to age-matched normal controls. Since the surgical inflammation that would not be present in a clinical CLI population, had largely subsided by day 14, two groups of CLI mice were treated with pFUS starting on day 14. One group received a single treatment of pFUS on day 14 and was euthanized 24 hr later (day 15); a second group received daily treatment of pFUS for 3 consecutive days (days 14, 15, and 16) and was euthanized 24 hr after the third pFUS treatment (day 17). We previously demonstrated that 3 daily courses of pFUS to skeletal muscle and MSC infusions led to greater MSC homing and therefore, would potentially use a similar protocol for CLI treatment^34^. Groups were compared to day 0 controls by ANOVA. The single pFUS treatment significantly increased expression (p < 0.05) of IL-1α, IL-1β, IL-4, IL-5, IL-10, IL-12p40, IL-12p70, IL-13, IL-17, G-CSF, KC, MCP-1, MIP-1α, FGF, VCAM and regulated on activation, normal T cell expressed and secreted (RANTES). Three daily courses of pFUS significantly (p < 0.05) increased IL-1α, IL-1β, IL-4, IL-5, IL-10, IL-12p40, IL-13, IL-15, IL-17, G-CSF, KC, MCP-1, MIP-1α, FGF, VCAM IL-1α, IL-4, IL-10, IL-15, FGF, RANTES, and vascular endothelial growth factor (VEGF) (Fig. 2, Supplemental Figure 1).

### MSC homing to pFUS treated ischemic muscle

**Figure 3:**
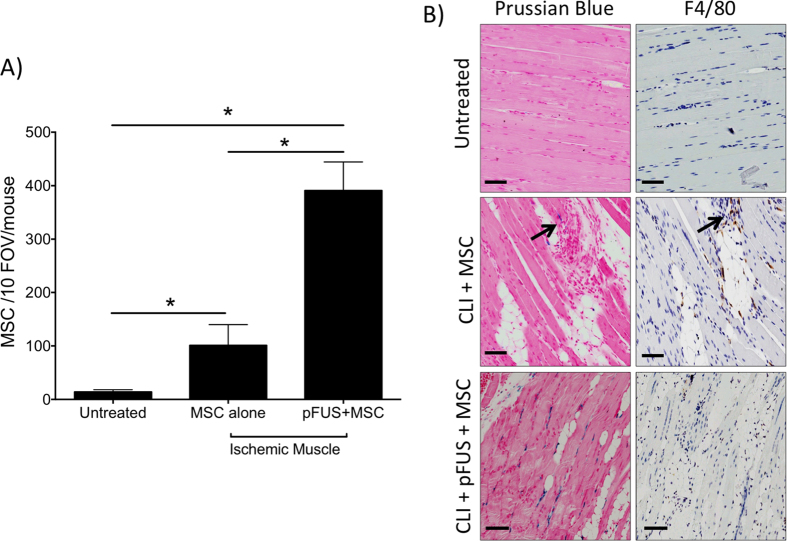
Mesenchymal stem cell (MSC) homing to critical limb ischemia CLI muscle with and without pulsed focused ultrasound (pFUS). Mice (n = 5 per group) were given CLI surgery on day 0 and then on day 14, superparamagnetic iron oxide nanoparticle (SPION)-labeled MSC were IV infused (10^6^ per animal) with and without pFUS. Mice were euthanized 24 hr after treatment (day 15) and MSC were counted. MSC were defined as cells positive for both F4/80 and Prussian blue (PB). (**A**) Statistically greater numbers of MSC homed to CLI muscle compared to healthy (untreated) muscle, but homing is further increased to CLI muscle using pFUS. *Denotes statistical significance (p < 0.05) by one-way ANOVA. (**B**) Representative microscopy showing PB^+^ cells (left, blue cells) and F4/80^+^ cells (right, brown cells) in each group. Scale bar = 200 μm.

Having demonstrated that post-surgical inflammation in CLI subsides by day 14, but that single or multiple courses of pFUS reinvigorate molecular responses in the CLI muscle, we investigated if IV-infused MSC homing would be increased to sonicated tissues. Two groups of mice were given 10^6^ human MSC labeled with superparamagnetic iron oxide nanoparticles (SPION) and one group received pFUS 1 hour after injection. Mice (n = 5 per group) were euthanized 24 hr post-injection and Prussian blue (PB) staining detected iron-labeled MSCs in the ischemic and normal contralateral hamstrings. Adjacent histological sections were immunostained with F4/80 (mouse macrophage marker) to identify macrophages that may have phagocytosed MSC, SPION released by apoptotic/dead MSC, or blood/tissue byproducts. Cells that were PB-positive and F4/80-negative were considered viable MSC and counted during analyses (Fig. 3). MSCs were counted in 10 high-power fields-of-view (FOV) per section in 3 sections per mouse. A statistically significant increase (p < 0.05) in MSC homing to non-pFUS-treated ischemic muscle (101 ± 39) was observed compared to MSC homing to healthy control muscle (14 ± 4 MSC). Additional increases in MSC homing were observed in CLI muscle treated with pFUS (391 ± 53) that were statistically significant compared to sham muscle or non-pFUS-treated CLI muscle from animals receiving MSC infusions (p ≤ 0.0001) (Fig. 3).

### Combining pFUS and MSC improves long-term perfusion of ischemic limbs

**Figure 4:**
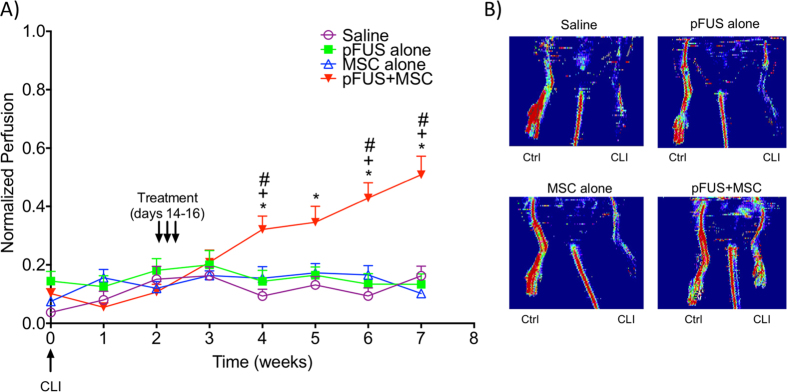
Weekly laser Doppler perfusion imaging (LDPI) in the feet of mice. (**A**) Mice had critical limb ischemia (CLI) induced on Week 0. Two weeks post-surgery, mice were treated for 3 consecutive days (Day 14, 15, and 16) with: saline (n = 8), pFUS (n = 7), mesenchymal stem cells (MSC 10^6^/day IV, n = 8) and pulsed focused ultrasound (pFUS) + MSC (10^6^/day IV, n = 17) and imaged by LDPI weekly for another 5 weeks (7 weeks post-surgery). Statistical significance (p < 0.05; two-way ANOVA) of the pFUS + MSC groups is indicated by * compared to the saline group, + compared to the MSC only group, # compared to the pFUS only group. (**B**) Representative LDPI of saline, pFUS, MSC, and pFUS + MSC at 7 weeks post induction of CLI.

Two weeks post-surgery, mice were divided into 4 groups and treated on days 14, 15, and 16 with IV saline (n = 8), pFUS alone (n = 8), IV MSC alone (10^6^/day; n = 8), or pFUS + MSC (10^6^/day, n = 17). Mouse feet were monitored by LDPI for 7 weeks (Fig. 4) and then euthanized for subsequent histological analyses. The number of mice in the pFUS + MSC group was increased to validate the perfusion changes observed in the original cohort (first cohort, n = 8; second cohort, n = 9). A group receiving only pFUS was included in this study because pFUS increased expression of CCTF and CAM. While these changes are harnessed to enhance MSC tropism, they could also influence CLI outcomes.

Beginning at 2 weeks post-treatment (4 weeks post-surgery), foot perfusion significantly increased in mice receiving pFUS + MSC compared to mice that received MSC alone (p < 0.05), pFUS alone (p < 0.05), or saline controls (p < 0.01) (two-way ANOVA). Perfusion in feet increased week-by-week in pFUS + MSC-treated limbs for the duration of the study (Fig. 4) and was approximately 50% of the contralateral foot perfusion at 7 weeks post-surgery. All other groups (saline, pFUS alone, or MSC alone) showed no statistically significant changes in perfusion throughout the entire 7-week study. MSC do release pro-angiogenic factors^12^ and MSCs did home to CLI muscle without pFUS, however MSC alone were insufficient to improve/reestablish perfusion. Similarly, pFUS alone did release anti-inflammatory and pro-angiogenic factors, but it is likely their expression was too short-lived to alter functional disease outcomes.

### Histological evaluation for capillary density and fibrosis

**Figure 5:**
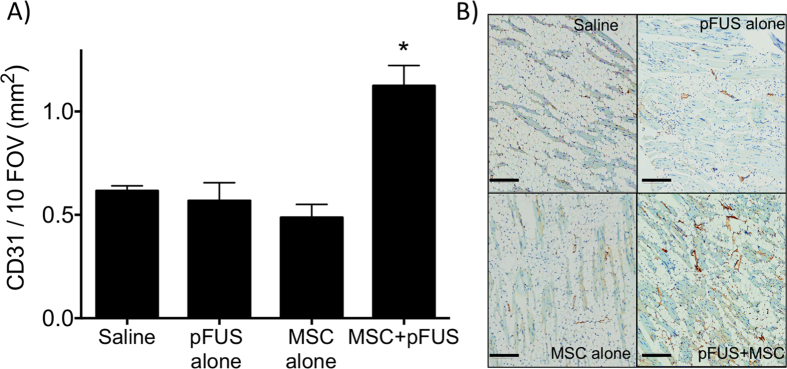
CD31 immunostaining in treatment groups 7 weeks post-surgery. (**A**) Area of CD31-positive signal for each treatment group. (n = 3 slides per animal; 5 animals per groups) *Indicates significance (p < 0.05) by one-way ANAOVA. (**B**) Representative images of CD31 staining with CD31 appearing in brown. Scale bar = 200 μm.

To further investigate perfusion differences between groups, muscle at 7 weeks post-surgery was subjected to immunohistochemistry for the endothelial cell marker CD31 to quantify vessel density (Fig. 5). CD31-positive cells were counted in 10 FOV per section using 3 sections per mouse (n = 5 mice per group). Significantly greater CD31 expression was observed in pFUS + MSC-treated CLI muscle compared to CLI muscle from the MSC alone (p ≤ 0.0001), pFUS alone (p ≤ 0.001) or saline groups (p ≤ 0.001).

**Figure 6:**
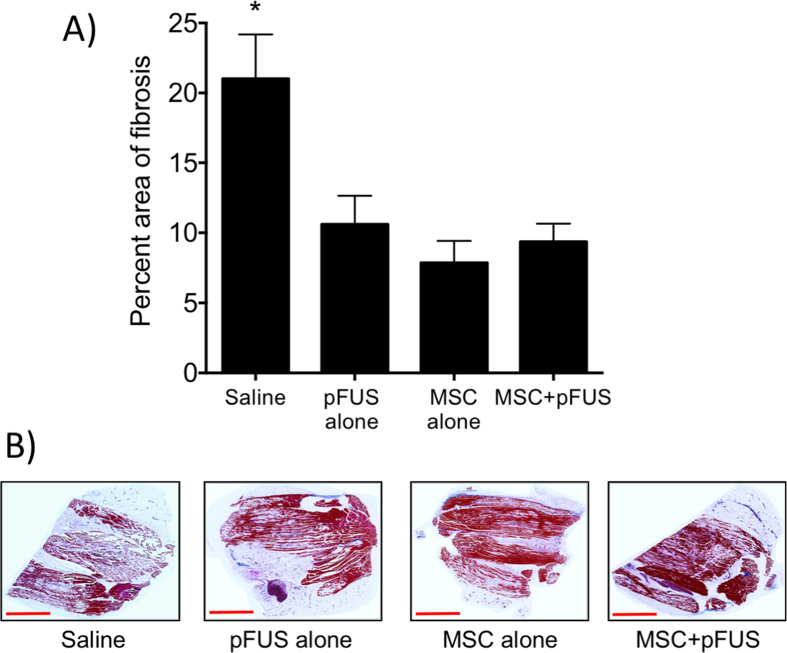
Masson Trichrome staining in treatment groups 7 weeks post-surgery. (**A**) Percentage area of fibrosis for each treatment group. (n = 3 slides per animal; 5 animals per groups) *Indicates significance (p < 0.05) by one-way ANOVA. (**B**) Representative images of Masson Trichrome stain. Scale bar = 3 mm.

To determine if there were differences in fibrotic tissue volumes in muscle at 7 weeks post-surgery, Masson trichrome staining was performed (Fig. 6). Fibrotic regions were segmented using Image J and their areas were calculated as a percentage of the total tissue area per section and results from all groups (n = 5 mice per group) were compared by ANOVA. The percentage of fibrotic area in saline-treated controls was 21%. The percentage of fibrosis decreased in the mice receiving pFUS alone (11%; p < 0.01), MSC alone (8%; p < 0.001), or pFUS + MSC (9%; p < 0.001).

### MSCs homing to pFUS-treated CLI muscle express more VEGF and IL-10

**Figure 7:**
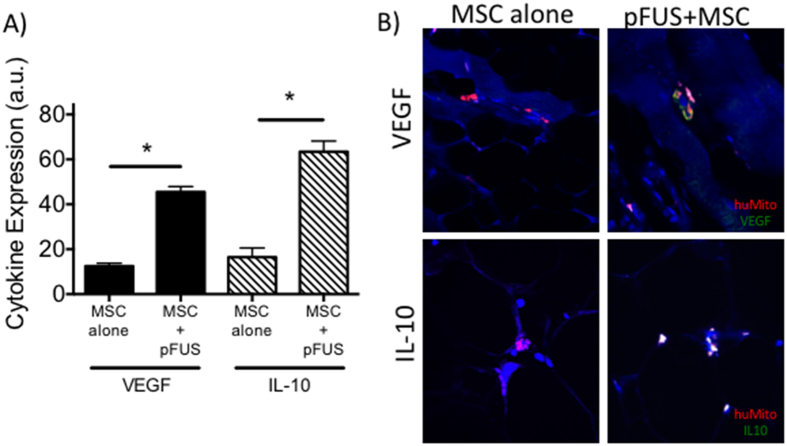
Immunostaining for human vascular endothelial growth factor (VEGF) and interleukin (IL)-10 reveals greater cytokine production by mesenchymal stem cells (MSC) in homing to muscle previously treated with pulsed focused ultrasound (pFUS). (**A**) Relative fluorescence intensity of IL-10 or VEGF in regions-of-interest (ROI) drawn around human MSCs (n = 10 ROI from 5 mice per group). *Indicates significance (p < 0.05) by one-way ANOVA with Bonferroni correction. (**B**) Representative fluorescence images showing human MSCs (red) and either VEGF or IL-10 expression (green).

While increased MSC homing to pFUS-treated CLI muscle could be responsible for improved outcomes, it is possible that the molecular changes occurring in the muscle after pFUS alter MSC function in addition to serving as chemoattractants that increase homing. At 14 days post-surgery, mice with CLI were treated with pFUS and MSCs or MSCs alone and euthanized at 24 hrs post-treatment. Immunofluorescence microscopy measured expression of human VEGF and human IL-10 (produced by MSC rather than host tissue) near or in MSCs. The human MSCs in the muscle of both groups of mice (n = 5 per group) were identified using an anti-human mitochondrial antibody. Identically sized region of interests (ROI) were drawn around human MSCs and the average fluorescence signal corresponding to VEGF or IL-10 in the ROI was measured. Background fluorescence was measured using isotype staining and subtracted from measurements. MSCs in CLI muscle that received pFUS showed a statistically significant (p < 0.001) 3-fold increase in VEGF expression, and a 4-fold increase in IL-10 expression (p < 0.0001) compared to MSC in CLI muscle that did not receive pFUS (Fig. 7).

## Discussion

The major findings of this study are: (a) primarily pro-inflammatory CCTF are increased in CLI muscle at 1 day post-surgery and return to baseline levels by 14 days, leaving a residual post-surgical effect of increased CAM expression; (b) at 14 days post-surgery, pFUS reinvigorated a molecular response in CLI muscle; (c) the pFUS molecular response could be harnessed to increase homing of IV-infused MSC to CLI hind limbs; (d) MSCs that homed to pFUS-treated muscle expressed more VEGF and IL-10 than MSC homing to non-pFUS-treated muscle; and (e) pFUS + MSC significantly increased foot perfusion and vascular density in ischemic hind limbs, but neither MSC infusions alone nor pFUS alone did so.

### Clinically relevant rodent CLI model

Many PAD patients are older and have diminished regenerative capacity^2^,^4^. However, appropriate preclinical models are infrequently used (Supplemental Table 2). Most preclinical models display spontaneous/endogenous reperfusion and do not allow long-term hypoperfusion^25^. Therefore, we used 10–12-month-old mice, corresponding to a human age 50–65 years old, which are less likely to demonstrate spontaneous reperfusion^38^. Second, we utilized EIA ligation and transection^39^, resulting in the ischemic limbs did not spontaneously reestablish blood flow, especially when combined with aged mice. Preclinical PAD/CLI models often receive cell therapy soon after injury—in the presence of acute post-surgical inflammation that typically does not exist in clinical patients. This could generate spuriously positive findings. Therefore, this study administered therapy after acute inflammation subsided.

### Proteomic changes from CLI and pFUS in muscle tissue

The inflammatory response (mainly IL-1 and TNFα) to ischemic insults has been shown to persist for up to 7 days^40^. We demonstrated that the increased expression of CCTF and CAM at day 1 post-surgery, consistent with other reports^41^,^42^, and elevations declined to normal values by day 14 with the exception of CAM. pFUS at day 14 post-surgery reinvigorated molecular responses that were characterized by pro- and anti-inflammatory cytokines and trophic factors. pFUS generated a similar proteomic profile in aged mice with CLI as it did in normal skeletal muscle from young C3H mice^35^. This profile contains a number of chemoattractants for MS and importantly, we demonstrated that pFUS increased CCTF and CAM outside of the acute inflammatory window associated with surgically-induced ischemia.

### pFUS induced MSC homing to ischemic limbs

pFUS increases CCTF and CAM in the targeted parenchyma to generate what we have termed a “molecular zip-code”^32^,^34^. The mechanical effects of pFUS induce release of chemoattractants that increases tropism of IV-infused stem cells to sonicated tissues^32^,^34^,^35^,^36^. Repeating courses of pFUS and MSC daily for 3 days maximized the magnitude of MSC homing in muscles^34^. In the current study, we showed that pFUS coupled with IV MSC at 14 days post-ischemia (when acute inflammation has subsided) resulted in greater numbers of MSC in targeted muscle compared to MSC injections alone. We observed few F4/80-PB^+^ macrophages in the ischemic limb, consistent with either the clearing of the initial cellular debris following surgery or minimal uptake of dead human MSC^43^. Further research will be required to determine if the amount and/or timing of the IV MSC infusion in this CLI model is appropriate to maximize MSC homing to ischemic muscle.

### pFUS and MSC therapy improved hindlimb perfusion in a CLI model

Three daily courses of pFUS with IV MSC increased vascular density in the hamstring and perfusion in the feet at 7 weeks post-treatment compared to three daily courses of MSC alone or pFUS alone. It is unlikely that infused MSC engraft and survive in the ischemic muscle over 7 weeks, but rather, they likely alter the parenchymal microenvironment and stimulate endogenous regenerative mechanisms. Lack of increased blood flow and vessel density in the MSC alone cohort was unanticipated, especially because of previous studies using MSC alone^20^,^21^ (Supplemental Table 1). It is unclear why MSC alone did not have significant treatment effects. One explanation is the greater severity of CLI and animal age in our model. Other studies focused on the femoral artery^18^,^20^,^21^,^44^,^45^ in young (i.e., 6–12 week old) mice^19^,^20^,^44^,^45^,^46^,^47^ that may not adequately model CLI. Many previous studies demonstrate spontaneous revascularization^48^,^49^. MSC therapies in previous studies may only be accelerating endogenous reperfusion in comparison to our model. Other potential explanations include the type of stem cell used (allogeneic, autologous, or xenograph), the route of administration (i.e., IV, intra-arterial [IA], or IM), and the timing of cell transplantation following surgical ligation. For example, studies using systemic cell infusion demonstrate increases in blood flow^13^,^45^,^47^, but infusions typically occur ≤24 hr post-surgery, during acute inflammation. Innate inflammation following surgery may be a unique complicating factor in these models that inadvertently enhances stem cell homing and function. By waiting for surgically induced inflammation to subside, MSC homing in our study (which was minimal) mirrors that which would occur clinically.

The significant differences in physiological and histological findings between the pFUS + MSC and MSC alone cohorts suggest that pFUS may precondition the muscle microenvironment to potentiate MSC. Numerous studies have demonstrated that pretreating MSC *in vitro* with pro-inflammatory cytokines increased production anti-inflammatory factors by MSCs, and some studies have shown improved outcomes in mouse graft-versus-host disease models^50^,^51^. We previously reported that pFUS to the renal parenchyma during AKI acts as an *in vivo* conditioning tool by upregulating renal IFNγ that stimulates production of IL-10 by MSC^32^. In the current study, we observed significantly greater production of human VEGF and IL-10 by MSCs that homed to pFUS-treated CLI muscle compared to MSCs that homed to untreated CLI muscle. This suggests that pFUS might act as a neo-adjuvant to MSC therapy in CLI by increasing cell potency. While human-specific antibodies were employed in this study, some cross-reactivity may have occurred and this finding will be validated by additional methods (e.g. RT-PCR). Furthermore, we plan to conduct loss-of-function studies where MSC expression of VEGF or IL-10 is silenced.

Although we did not perform a time course analysis of CCTF following pFUS to ischemic muscle (the only measurement was performed 24 hr post-pFUS), it is possible that several molecular factors could have been elevated at earlier time points^32^,^33^,^34^,^35^,^36^. While undetected by us, these could alter the function of infused MSC. Moreover, investigating murine cytokine profiles in the different treatment groups could further reveal targets upon which human MSC alter and illuminate mechanisms of action for MSC in CLI.

There are several limitations of this study. LDPI can monitor perfusion changes in the feet and calves, but not deep structures. However, hamstrings were necessary to be targeted by pFUS to obtain sufficient material for molecular analyses; therefore, we were unable to image perfusion in the exact region that was sonicated. Additional clinical manifestations such as limb loss were not characterized in this study, but will be included in followup reports. The unbalanced numbers of mice in the different cohorts may have contributed to a possible bias in favor of the pFUS + MSC group, however when analyses were performed with equal numbers of animals in each cohort the perfusion and histological results were unchanged. Lastly, we did not explore the possibility of IM or IA injections into the ischemic hindlimb that are common routes of administration for cellular therapy in clinical and experimental PAD studies^25^. Direct implantation of MSC has several advantages including increasing the local production of anti-inflammatory and angiogenic factors in the ischemic limb that contribute to increased vessel density. With IM injections however, cells remain localized near the site of injection. The procedure requires multiple implantation sites and that clinical subjects be anesthetized due to dysesthesia. Further research should determine if pFUS would alter function of MSC administered IM or IA and if some combination of administration routes in addition to pFUS would be optimal.

### Implications of the study

The combination of pFUS and MSC infusions could have therapeutic value for PAD patients with the most severe forms of vascular insufficiency who have not improved following lifestyle changes or pharmaceutical treatment. pFUS is image-guided, noninvasive, and nondestructive. With appropriate oversight from regulatory authorities, it is easily translatable to the clinic using currently-approved instrumentation. Moreover, sophisticated therapeutic paradigms can be developed with pFUS and stem cell infusions since this approach can be repeated multiple times and at multiple locations. Clinical trials are needed to determine the effectiveness of cellular therapy in PAD, but it is possible the combination of cell delivery methods with pFUS serving as a neoadjuvant might lead to more reliable and durable clinical outcomes.

## Conclusion

Image-guided pFUS is a clinically relevant approach to enhance homing, permeability, and retention of IV-infused MSC and restore perfusion in ischemic hindlimbs. After waiting for acute surgical inflammation to subside, mechanical pFUS effects stimulated local molecular changes in the tissue microenvironment to enhance homing of infused MSC. Furthermore, pFUS appears to molecularly condition CLI muscle in a way that improves MSC potency, resulting in better reestablishment of perfusion than MSC infusions alone. This proof-of-concept study experimentally models the smoldering inflammation that is associated with the gradual and often insidious onset of PAD. Furthermore, it demonstrates that pFUS with stem cell infusions enhances clinical and functional CLI outcomes when administered outside of the acute inflammatory window associated with ischemic insults.

## Materials and Methods

### Animals

Animal studies and protocols were approved by the National Institutes of Health Clinical Center Animal Care and Use Committee. All methods and procedures were performed in accordance with the relevant guidelines and regulations. Female C3H (Charles River Laboratories, Wilmington, MA) 10–12 months old were used for studies. Mice were anesthetized with 1.5–2.5% isoflurane in O_2_.

### Critical Limb Ischemia

Mice underwent unilateral external iliac artery ligation and cauterization in the right leg. A longitudinal incision was made with Metzenbaum scissors from the inguinal area to the proximal stifle. The external iliac nerve, artery, and vein were isolated and two ligatures (6–0 nylon) were tied around the external iliac artery before cauterization. Skin was sutured and treated with antibiotic ointment. Buprenorphine SR-Lab (ZooPharma, Windsor, CO) was given subcutaneously at 1 mg/kg after surgery and as needed thereafter.

### pFUS Sonication

A VIFU 2000 (Alpinion, Bothell, WA) was used at a frequency of 1 MHz. Hamstrings were treated in degassed water at 37 °C using a 4 × 5 matrix (spacing = 2 mm). Each point received 100 pulses with the following parameters: acoustic power, 40 W; pulse repetition frequency, 5 Hz; and duty cycle, 5% (10 ms ON; 190 ms OFF). Control mice received sham pFUS exposures (transducer power = 0 W).

### MSC culture and infusions

Human MSCs (NIH Center for Bone Marrow Stromal Cell Transplantation) were culture-expanded in α-minimum essential medium (Life Technologies, Carlsbad, CA) with 20% fetal bovine serum (Gemeni Bio-products, Sacramento, CA) at 37 °C under 5% CO_2_. MSCs were incubated with super-paramagnetic iron oxide nanoparticles (SPION) for histological detection. MSCs were detached with TrypLE (Life Technologies, Carlsbad, CA) and suspended at 10^7 ^cells/mL in Hank’s Balanced Salt Solution (Life Technologies) containing 10 U/mL Heparin (sodium salt) (Hospira, Lake Forest, IL). MSC (1 × 10^6^ in 100 μL) were injected into the tail vein ~1 hr before pFUS.

### Perfusion Imaging

Mice were imaged for ~10 min using a Laser Doppler imager (Moor, Axminster, UK). Images were analyzed with MoorLDI Image Review (V5.3). Regions-of-interest (ROI) were drawn around the legs and perfusion ratios of the ischemic:non-ischemic limbs were calculated^16^,^52^.

### Tissue harvesting

Mice (n = 6 per group) were euthanized at various time points and pFUS-treated and untreated contralateral hamstrings for molecular analyses were frozen in liquid N_2_. Separate cohorts of mice (n = 5 per group), were perfused with heparanized-saline followed by ice-cold phosphate buffered saline (PBS) containing 4% paraformaldehyde (PFA). Dissected hamstrings were then fixed in 4% PFA for 24 hr and maintained in PBS until samples were embedded in paraffin.

### Proteomic Analyses (Cytokines, Chemokines, Trophic Factors, and Cell Adhesion Molecules)

Frozen muscle was homogenized in cell lysis buffer (Cell Signaling Technology, Danvers, MA) containing protease inhibitor (Santa Cruz Biotechnoloy, Santa Cruz, CA). Samples were centrifuged at 14000 rpm for 20 minutes at 4 °C and supernatants were used for analyses. Total protein was determined using a bicinchononic acid (BCA) assay (Thermo Scientific, Waltham, MA). Homogenates (2 mg/mL total protein) were analyzed by multiplex ELISA (Bio-Plex Pro Mouse 23-Plex and 9-Plex assays, Bio-Rad, Hercules, CA) kits using a Bio-Plex 200 (Bio-Rad). Hepatocyte growth factor (HGF), ICAM-1 and VCAM-1 were analyzed by ELISA (RayBiotech, Inc., Norcross, GA) with a protein concentration of 1.25 mg/mL and read on a spectrophotometric plate reader (Spectra Max M5, Molecular Devices, Sunnyvale, CA). All assays were performed according to manufacturer instructions.

### Histological staining analyses

Prussian blue staining was used to detect iron-labeled MSC by incubating slides in 10% potassium ferrocyanide and 10% hydrochloric acid for 30 min. Slides were washed extensively in deionized water and counterstained with Nuclear Fast Red (Scytek, Logan, UT) for 3 min. MSCs were defined as Prussian-blue-positive, F4/80-negative (see protocol below) cells that were counted in 10 high power fields-of-view (FOV) per section in 3 sections per mouse. Fibrosis was detected using a Masson’s Trichrome staining kit (#HT-15, Sigma-Aldrich, St. Louis, MO) according to the manufacturer’s protocol. Fibrotic tissue and muscle tissue were segmented using ImageJ (NIH) and fibrosis was expressed as the percentage of fibrotic tissue in each section.

IHC was performed to detect F4/80, CD31, human mitochondria, human VEGF, and human IL-10. Sections were blocked using Super Block (Thermo Scientific, Waltham, MA) for 10 min and incubated with the following antibodies (dilutions in parentheses): rat anti-F4/80 (1:100); rabbit anti-CD31 (1:200); mouse anti-mitochondria (1:200); rabbit anti-human IL-10 (1:50); or rabbit anti-human VEGF (all antibodies from Abcam, Cambridge, MA) overnight at 4 °C. Species-appropriate secondary antibodies that were conjugated to horseradish peroxidase (HRP), DyLite 550, or DyLite 650 were used for detection. CD31^+^ endothelial cells were identified using 3,3′-diaminobenzidine (DAB) (Sigma-Aldrich, St. Louis, MO). An individual blinded to which groups sections were obtained performed bright-field imaging and counting of human MSC.

### Microscopy

Bright-field microscopy was performed with an Aperio ScanScope CS equipped with a 20× air objective (NA = 0.75, Leica Microsystems, Buffalo Grove, IL).

### Statistical Analyses

Data are presented as mean ± SEM. Statistical analyses were performed with Prism (GraphPad Inc., La Jolla, CA). Student’s *t*-tests were used for pairwise comparisons and one- or two-way analysis of variance (ANOVA) with Bonferroni post-hoc tests were used for multiple comparisons. P-values < 0.05 were considered statistically significant.

## Additional Information

**How to cite this article**: Tebebi, P. A. *et al*. Improving the therapeutic efficacy of mesenchymal stromal cells to restore perfusion in critical limb ischemia through pulsed focused ultrasound. *Sci. Rep.* **7**, 41550; doi: 10.1038/srep41550 (2017).

**Publisher's note:** Springer Nature remains neutral with regard to jurisdictional claims in published maps and institutional affiliations.

## Supplementary Material

Supplementary Tables and Figures
