# Supplementary material for: Improving the therapeutic efficacy of mesenchymal stromal cells to restore perfusion in critical limb ischemia through pulsed focused ultrasound
Source: Sci Rep. 2017 Feb 7;7:41550. doi: 10.1038/srep41550 (PMC5294408; doi:10.1038/srep41550)
Supplement: Supplementary Tables and Figures [file srep41550-s1.pdf]

Supplemental Data and information for:

Improving the therapeutic efficacy of mesenchymal stromal cells to restore perfusion in critical limb ischemia through pulsed focused ultrasound

Pamela A. Tebebi[1,2], Saejeong J. Kim[1], Rashida A. Williams[1], Blerta Milo[1], Victor Frenkel[3], Scott R. Burks[1], and Joseph A. Frank[1,4]

[1]Frank Lab, Radiology and Imaging Sciences Dept., Clinical Center, National Institutes of Health, Bethesda, Maryland, USA, [2]Department of Biomedical Engineering, Catholic University of America, Washington, DC, USA, [3]Department of Diagnostic Radiology and Nuclear Medicine, University of Maryland School of Medicine, Baltimore, MD, USA and [4]National Institutes of Biomedical Imaging and Bioengineering, National Institutes of Health, Bethesda, Maryland, USA

Corresponding Author
Joseph Frank M.S. M.D.
Building 10 Room B1N256
10 Center Drive MSC 1074
Bethesda, Maryland 20892-1074
phone 301-402-4314
fax 301-402-3216
jfrank@helix.nih.gov

Supplemental Table 1: List of Abbreviations

bFGF	Basic Fibroblast Growth Factor
BMSC	Bone Marrow Stromal Cells
CAM	Cell Adhesion Molecules
CCTF	Cytokines, Chemokines, Trophic Factors
FUS	Focused Ultrasound
GCSF	Granulocyte Colony-Stimulating Factor
GMCSF	Granulocyte Macrophage Colony-Stimulating Factor
HSP-70	Heat Shock Protein 70
ICAM	Intercellular Adhesion Molecule
IFNγ	Interferon Gamma
IGF-1	Insulin-like Growth Factor
IL1α	Interleukin-1 Alpha
IL1β	Interleukin-1 Beta
IL2	Interleukin-2
IL3	Interleukin 3
IL4	Interleukin 4
IL5	Interleukin 5
IL6	Interleukin 6
IL9	Interleukin 9
IL10	Interleukin 10
IL12p40	Interleukin 12 (p40 subunit)
IL12p70	Interleukin 12 (p70 subunit)
IL13	Interleukin 13
IL15	Interleukin 15
IL17	Interleukin 17
IL18	Interleukin 18
KC	Keratinocyte-derived Chemokine or Keratinocyte Chemoattractant
LIF	Leukemia Inhibitory Factor
MSC	Mesenchymal Stem Cell
MCP1	Monocyte Chemoattractant Protein-1
MCSF	Monocyte Colony Stimulating Factor
MIG	Monokine Induced by Gamma-Interferon
MIP1α	Macrophage Inflammatory Protein 1 Alpha
MIP1β	Macrophage Inflammatory Protein 1 Beta
MIP2	Macrophage Inflammatory Protein 2
NFκB	Nuclear Factor Kappa-Light-Chain-Enhancer of Activated B Cells
PDGF	Platelet-Derived Growth Factor
pFUS	Pulsed Focused Ultrasound
RANTES	Regulated on Activation, Normal T-Cell Expressed and Secreted
SCF	Stem Cell Factor

SDF-1α	Stromal Cell-Derived Factor 1 Alpha
TNFα	Tumor Necrosis Factor Alpha
VCAM	Vascular Cell Adhesion Molecule
VEGF	Vascular Endothelial Growth Factor

Supplemental Table 2: Pre-clinical PAD studie

Ref #	Animal Model	Age	Ischemia Model	Cell type	Route of admin.	Time of Cell Admin Post-PAD	Outcome- first day increase in perfusion
45	C57BL/6 m; $CD4^{-/-}$ m	12wk	Acute	$mCD4^{+}$ cells, mCD4 depleted spleenocytes	IV	Day 3	Day 28
48	Athymic nude m	8-10wk	Critical	CM-Dil-labeled hEPC	IV	Day 0	Day 21
46	Nude KSN/Slc m	8wk	Acute	h-VPC	IA	Day -1	Day 14
N/A *	Immunodeficient m	NR	Critical	hBM-EC, hiPSC-EC, hESC-CE	IM	Day 0	Day 14
18	Syngenic FVB m	10-12wk	Acute	mMNC	IM IM	Day 0	NS
	C57BL/6 m	10-12wk	Acute	mMNC	IV	Day 0	NS
15	Athymic nude r	5 wk	Acute	hEPC + SW	IV	3wk	2 wk post treatment
19	C57BL/6 m	6-8 wk	Acute	mMNC	IM	Day 0	Day 7
16	Athymic nu r	8wk	Acute	hEPC	IM	Day28 Day 31 Day 34	Day 42
20	C57BL/6J m	8wk	Acute	mMSC + Simvastatin (for 21 days)	IM	Day 0	Day 21
17	Fisher F344 r	NR	Acute	rEPC; UM-SDF-1 + rEPC	IV	Day 14	Day 28
13	NOD/SCID β2M null or NOD/SCID/MPSVII m	NR	Acute	hMNC, ALDHlo, ALDHhi cells, $CD14^{+}$ cells	IV	Day 0-1	Day 7

Reference	Animal Model	Age	Ischemia Model	Cell type	Route	Time of Cell Admin Post-PAD	Outcome-initial day of significant increase in perfusion
47	C nude m	8-10wk	Critical	mADSC	IM	Day 1	Day 14
21	C57BL/6J m	NR	Acute	mMSC	IM	Day 0	Day 7
22	C57BL/6J m	12-16wk	Acute	mMAPC-U, mBMC, mMAPC-VP	IM	Day 0	Day 4
	BALB/C-nu/nu	12-16wk	Critical	mMAPC-U, mBMC-U2, hMAPC-VP	IM	Day 5	Day 9
14	ApoE$^{-/-}$	8-15m	Acute	mBMC, mCD34^{+}/Mcad^{+} BMC mCD34^{-}/Mcad^{+} BMC	IA	Day 0	Day 0

* Lai, W.H. *et al*. PLoS One 2013;8:e57876

m - mouse; h - human; r - rat; rb - rabbit; nu - nude; IM - intramuscular; IV- intravenous; IA - intra-arterial; EP - electroporation; CM - conditioning media; NR-not reported; NS-not significant; SW-shock wave; wk- week(s); admin-administration, MSCs – mesenchymal stem cell, EPC – endothelial progenitor cell, EPC – endothelial progenitor cell, EC – endothelial-like cell, EC – endothelial-like cell, iPSC – induced pluripotent stem cell, BM - bone marrow mononuclear cells, ESC– embryonic stem cells, MNC – bone marrow– derived mononuclear cells, MSC – mesenchymal stem cells, UM – ultrasound-mediated, SDF-1 – stromal cell derived factor-1, lo – low, hi- high, ALDH – aldehyde dehydrogenase, ADSC – adipose-derived stromal cell, MAPC – multipotent adult progenitor cells, MAPC-U – undifferentiated MAPCs, MAPC-VP – MAPC-derived vascular pro-genitors, MAPC – derived vascular pro-genitors, BMC – bone marrow stem/progenitor cells, ApoE$^{-/-}$ – hypercholesterolemic mouse, CD34^{+}/Mcad^{+} BMC – BMC expressing both CD34 and M- cadherin surface molecules, CD34^{-}/Mcad^{+} BMC – BMC expressing M- cadherin surface molecules, CD34^{+}/Mcad^{-} BMC – BMC expressing both CD34 surface molecules

Supplemental Figure 1: Primary data of proteomic responses to critical limb ischemia (CLI) and pulsed focused ultrasound (pFUS). *p <0.05 using one-way ANOVA with Bonferroni correction; n=6 mice/group. Data are means ± SD. See Supplemental Table 1 above for abbreviations.

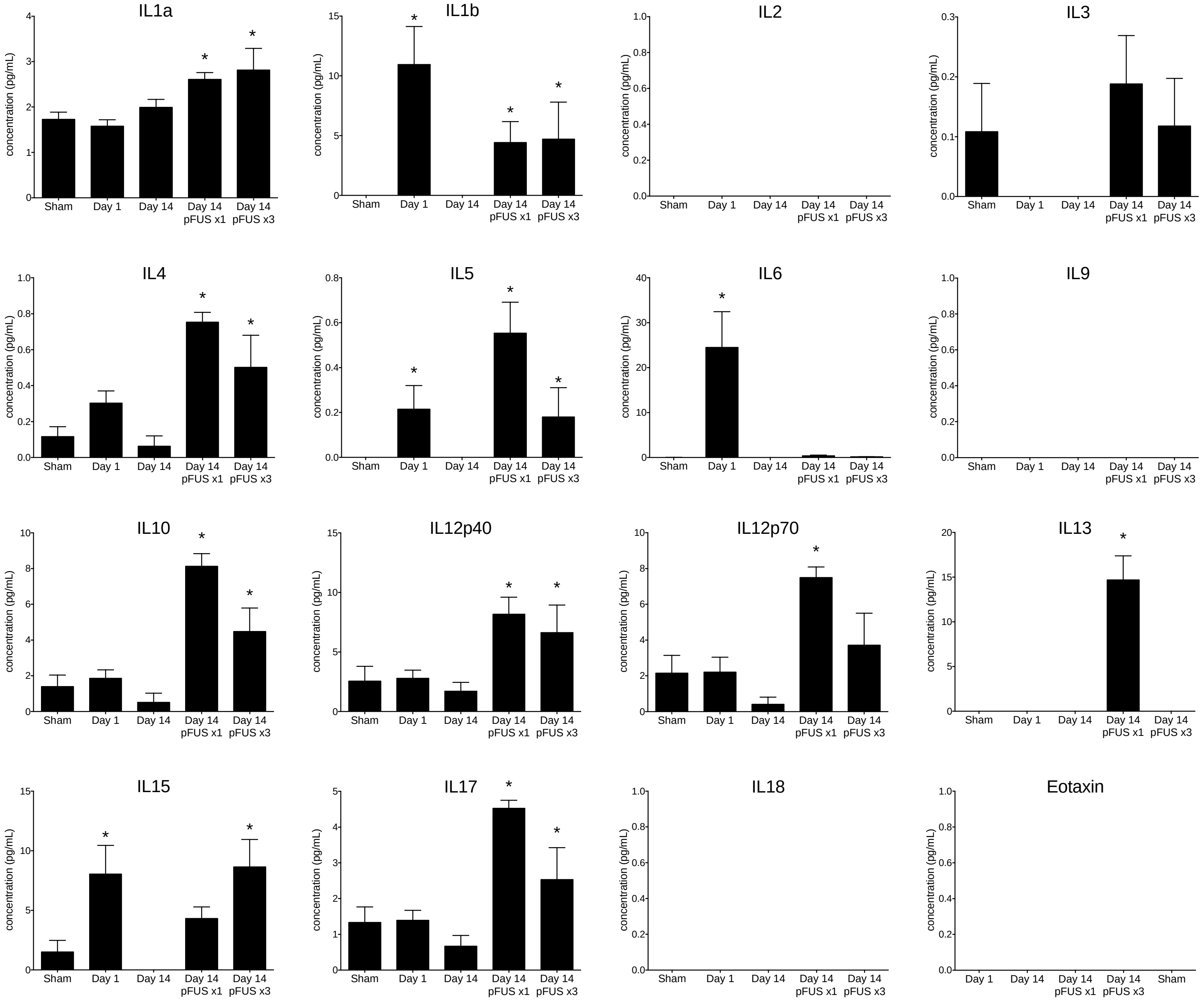

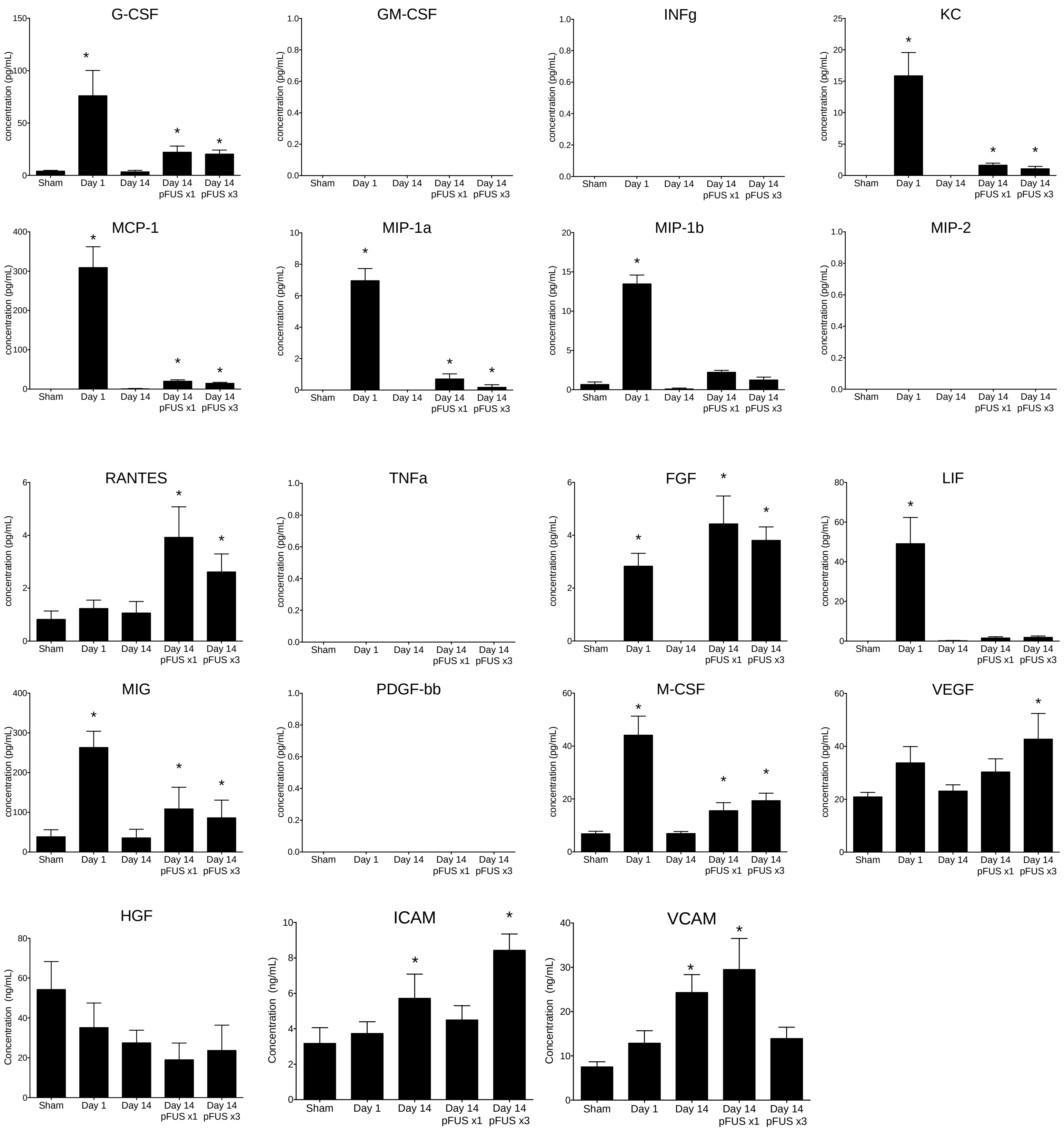

G-CSF
GM-CSF
INFg
KC
MCP-1
MIP-1a
MIP-1b
MIP-2
RANTES
TNFa
FGF
LIF
MIG
PDGF-bb
M-CSF
VEGF
HGF
ICAM
VCAM
concentration (pg/mL)
Concentration (ng/mL)
Sham
Day 1
Day 14
Day 14 pFUS x1
Day 14 pFUS x3
